# Leukocytosis Associated With Medroxyprogesterone in Gender-Affirming and Fertility Treatments

**DOI:** 10.7759/cureus.78681

**Published:** 2025-02-07

**Authors:** Salman Syed, Garry G Lachhar, Fatimah Spall, Alexandros Konstantinidis, David Chu

**Affiliations:** 1 Internal Medicine, Northwell Health, New York City, USA; 2 Hematology and Oncology, Northwell Health, New York City, USA; 3 Hematology and Oncology, Mather Hospital, New York City, USA

**Keywords:** gender-affirming care, hematology laboratory, internal medicine and endocrinology, pathology work up for targeted therapy, persistent leukocytosis

## Abstract

Leukocytosis, defined as an elevated white blood cell (WBC) count, can arise from physiological, infectious, neoplastic, or medication-related causes. While leukocytosis has been well-documented in conditions such as infections and malignancies, drug-induced leukocytosis is relatively rare, particularly in individuals undergoing gender transition therapies. Medroxyprogesterone, a progestin used in both fertility treatments and gender-affirming care, has been associated with hematologic changes, though its role in sustained leukocytosis remains underexplored. This case suggests a possible link between medroxyprogesterone and leukocytosis in gender-affirming therapy. It emphasizes the importance of thoroughly reviewing medication history to avoid unnecessary testing and procedures while highlighting the need for further research into the mechanism of progestin-induced hematologic changes.

## Introduction

Leukocytosis, characterized by an elevated white blood cell (WBC) count above 11,000/µL, is commonly associated with infections, inflammation, malignancies, and certain hematologic disorders [1]. However, chronic leukocytosis without a clear infectious or neoplastic cause can be challenging to diagnose. In such scenarios, the contribution of medications must be carefully explored. Although the hematologic side effects of steroids and some psychotropic drugs are well known, the role of medroxyprogesterone, a synthetic progestin used in gender-affirming and fertility treatments, remains underrecognized [2].

Gender-affirming therapy for transgender women typically involves estradiol, with or without adjunctive progestins like medroxyprogesterone. While these therapies aim to promote feminization, they may also have unintended side effects, including metabolic and hematologic changes. Few case reports and studies have explored the hematologic impact of these medications, particularly on chronic leukocytosis. Here, we present a case of persistent leukocytosis in a young transgender female undergoing gender transition therapy. This report underscores the importance of including medication-induced leukocytosis in the differential diagnosis and aims to contribute to the growing literature on hematologic side effects in the transgender population [3].

The objective is to discuss the diagnostic workup, differential diagnoses, and implications for management in such complex cases.

## Case presentation

A 20-year-old transgender female with a history of schizoaffective disorder and multiple prior suicidal attempts was admitted to the hospital due to suicidal ideation. During her hospitalization for psychiatric care, the hematology and medical oncology team was consulted to evaluate leukocytosis detected on admission, with a white blood cell (WBC) count of 20,000/µL.

The patient reported that her primary care physician was aware of her persistent leukocytosis, but no hematologic follow-up had been pursued. A review of outpatient records revealed that the elevated WBC count had persisted for nearly two years. She denied any B symptoms, including fevers, night sweats, or unintentional weight loss, as well as other systemic complaints such as fatigue, generalized weakness, headaches, lymphadenopathy, or pain.

As part of her male-to-female transition, the patient followed closely with an endocrinologist and has undergone bilateral orchiectomy. Her current medications include estradiol (1 mg, three tablets daily), medroxyprogesterone (5 mg, once daily), and lurasidone (40 mg, once daily). The patient denied chronic steroid use, alcohol consumption, smoking, or any illicit drug use. There was no reported history of drug allergies. The family history was non-contributory except for maternal iron deficiency anemia, which required oral iron supplementation.

A review of systems revealed no active complaints at the time of presentation. During the physical examination, the patient’s vital signs were stable (Table 1).

**Table 1:** Laboratory and diagnostic workup

Test/Investigation	Test/Investigation	Reference Range
White blood cell count (WBC)	20,000/µL	4,000-11,000/µL
Absolute neutrophil count (ANC)	11.3 × 10^9/L	1.5-8.0 × 10^9/L
Hemoglobin	12.3 g/dL	12-15 g/dL
Platelet count	378,000/µL	150,000-400,000/µL
Iron studies	Normal	-
Erythrocyte sedimentation rate (ESR)	5 mm/hr	0-20 mm/hr
C-reactive protein (CRP)	1 mg/L	0-3 mg/L
Urinalysis	Negative	-
Urine culture	Negative	-
Drug screening	Negative	-
Respiratory viral pathogen panel	Negative	-
Thyroid function tests	Normal	-
Leukocyte alkaline phosphatase (LAP) score	60	40-130
BCR-ABL fusion protein test	Negative	-
Chest X-ray	Negative	-

After optimizing psychiatric medication, the patient was on lurasidone, which was the home medication, and was additionally placed on trazodone and propranolol; the patient was discharged to follow up with the hematology-oncology outpatient clinic.

## Discussion

The persistent leukocytosis observed in this patient lasted nearly two years and prompted a comprehensive evaluation to exclude common causes such as infections, inflammatory conditions, and hematologic malignancies [4]. Extensive testing, including testing for myeloproliferative neoplasms and marrow or lymphoproliferative disorders, ruled out systemic pathology, turning attention toward medication-induced hematologic changes. This case underscores the need to recognize the potential of medroxyprogesterone acetate (MPA), a synthetic progestin, to induce sustained leukocytosis [5].

Medroxyprogesterone and hematologic mechanisms

Medroxyprogesterone, frequently used in contraception, hormone replacement therapy, and gender-affirming care, is primarily recognized for its effects on reproductive and metabolic systems. However, its hematologic impact remains underexplored. Current evidence suggests that progestins, including MPA, may alter hematopoiesis through several potential mechanisms [6].

Bone marrow stimulation

Progestins have been shown to stimulate hematopoietic progenitor cells, particularly those of the myeloid lineage, leading to increased production of neutrophils. Song et al. (1996) demonstrated that high-dose progestins can enhance white cell production and alter the cellular environment in target tissues, including the endometrium [7]. While the magnitude of leukocytosis induced by progestins is less dramatic than stress-related responses caused by glucocorticoids, it is nonetheless significant. The absence of a left shift or immature forms in the patient’s peripheral smear aligns with this mechanism, reflecting steady bone marrow stimulation without an acute or reactive process.

Anti-inflammatory and immunomodulatory effects

MPA exhibits partial agonist activity at glucocorticoid receptors, which may contribute to immunomodulation and subtle shifts in leukocyte production. While glucocorticoids are known to induce a rapid increase in circulating neutrophils by demargination and delayed apoptosis, MPA likely exerts a more gradual effect. Ito et al. (2014) highlighted that MPA enhances monocyte-endothelial interactions via upregulation of adhesion molecules, a phenomenon that could contribute to leukocyte redistribution and activation. Although primarily studied in vascular systems, these findings suggest that MPA may exert broader immunologic and hematopoietic effects [8].

Hormonal interactions in gender-affirming therapy

The concurrent use of estradiol and MPA in gender-affirming therapy creates a complex hormonal milieu. Estradiol influences inflammatory and metabolic pathways, modulating cytokine production and immune cell dynamics. Although estradiol alone has not been strongly associated with leukocytosis, its interplay with MPA may amplify progestin-induced effects on hematopoiesis. For example, estradiol can enhance vascular permeability and tissue remodeling, which might synergize with MPA’s immunomodulatory effects to create sustained hematologic shifts.

Evidence supporting medroxyprogesterone’s role in leukocytosis

Findings from this case are consistent with prior studies suggesting MPA’s potential to influence leukocyte dynamics. Song et al. (1996) provided foundational evidence of progestin-induced bone marrow activation, while Ito et al. (2014) demonstrated MPA’s capacity to alter immune cell behavior at the endothelial interface [7,8]. Additionally, research into depot formulations of medroxyprogesterone (DMPA) used in contraception has reported mild increases in WBC counts, further supporting the drug’s systemic impact on leukocyte populations [9].

The normal inflammatory markers and absence of systemic symptoms in this patient are notable and suggest that MPA-induced leukocytosis is unlikely to represent a pathologic immune response. Instead, the findings align with a steady-state hematopoietic stimulation, underscoring the need for heightened awareness of medication-induced hematologic changes in patients receiving long-term hormone therapy [10].

Differential diagnosis

The diagnostic workup effectively ruled out alternative causes of leukocytosis. A normal peripheral smear and inflammatory markers, along with the absence of systemic symptoms, excluded infectious and inflammatory conditions. Similarly, hematologic malignancies, including chronic myeloid leukemia, were ruled out with a normal leukocyte alkaline phosphatase (LAP) score and negative BCR-ABL test. Psychiatric medications like lurasidone were unlikely contributors, given their lack of established hematologic side effects. These findings point strongly toward medroxyprogesterone as a plausible etiologic factor in this case.

## Conclusions

This case underscores the critical importance of thorough medication history reviews in transgender patients undergoing gender-affirming therapy, particularly to recognize potential hematologic effects like leukocytosis induced by medications such as medroxyprogesterone acetate (MPA). Awareness of this phenomenon can prevent unnecessary diagnostic procedures and guide appropriate clinical management. Persistent leukocytosis, though not requiring immediate intervention in this case, highlights the need for continued monitoring to assess potential long-term implications. Further research into the hematologic effects of MPA within gender-affirming therapy is essential to optimize patient care.
